# The effect of a Housing First intervention on primary care retention among homeless individuals with mental illness

**DOI:** 10.1371/journal.pone.0246859

**Published:** 2021-02-11

**Authors:** Adam Whisler, Naheed Dosani, Matthew J. To, Kristen O’Brien, Samantha Young, Stephen W. Hwang

**Affiliations:** 1 MAP Centre for Urban Health Solutions, Li Ka Shing Knowledge Institute, St. Michael’s Hospital, Toronto, Ontario, Canada; 2 Inner City Health Associates, Toronto, Ontario, Canada; 3 Department of Family & Community Medicine, St Michael’s Hospital, Toronto, Ontario, Canada; 4 Division of Palliative Care, Faculty of Health Sciences, McMaster University, Hamilton, Ontario, Canada; 5 Division of Palliative Care, William Osler Health System, Brampton, Ontario, Canada; 6 Division of General Internal Medicine, Department of Medicine, University of Toronto, Toronto, Ontario, Canada; The MetroHealth System and Case Western Reserve University, UNITED STATES

## Abstract

**Background:**

Primary care retention, defined as ongoing periodic contact with a consistent primary care provider, is beneficial for people with serious chronic illnesses. This study examined the effect of a Housing First intervention on primary care retention among homeless individuals with mental illness.

**Methods:**

Two hundred individuals enrolled in the Toronto site of the At Home Project and randomized to Housing First or Treatment As Usual were studied. Medical records were reviewed to determine if participants were retained in primary care, defined as having at least one visit with the same primary care provider in each of two consecutive six-month periods during the 12 month period preceding and following randomization.

**Results:**

Medical records were obtained for 47 individuals randomized to Housing First and 40 individuals randomized to Treatment As Usual. During the one year period following randomization, the proportion of Housing First and Treatment As Usual participants retained in primary care was not significantly different (38.3% vs. 47.5%, p = 0.39). The change in primary care retention rates from the year preceding randomization to the year following randomization was +10.6% in the Housing First group and -5.0% in the Treatment As Usual group.

**Conclusion:**

Among homeless individuals with mental illness, Housing First did not significantly affect primary care retention over the follow-up period. These findings suggest Housing First interventions may need to place greater emphasis on connecting clients with primary care providers.

## Introduction

Homelessness is a complex health and social problem. In Toronto, the largest city in Canada, there are over 8,700 people who are homeless on any given night and approximately 19,000 unique individuals who use homeless shelters every year [1, 2]. When compared with the general population, homeless individuals have poorer health status and experience a high prevalence of physical and mental health problems [3–5]. About one-third of the homeless population reports a previous diagnosis of mental illness [6]. Due to these serious health needs, access to primary care is essential for homeless individuals. In Toronto, however, less than half of homeless adults report having a family physician [7].

For individuals with serious chronic illnesses, retention in primary care, defined as ongoing periodic contact with a consistent primary care provider (PCP), may be as important as simply having an identifiable PCP. The beneficial effect of retention in primary care has been well documented in vulnerable populations such as individuals with HIV infection. In these studies, retention has often been defined as having one or more visits to a single care provider in each of two consecutive six-month periods [8, 9]. Research has demonstrated that retention in primary care is associated with increased survival in adults who are living with HIV [10, 11]. Retention in care has been shown to be beneficial for individuals seeking addictions management [12]. Engaging and retaining homeless individuals in primary care is an important concern given the prevalence of physical and mental health needs, lack of access to primary care and high frequency of emergency department visits in this population [6, 7, 13].

With regards to primary care needs, homeless individuals with mental illness experience a variety of physical health conditions including hypertension, diabetes, respiratory illnesses, skin infections, and malnutrition [14]. These individuals report physical disabilities such as impaired vision and mobility [15]. Smoking, alcohol and substance use disorders are also common among homeless individuals with mental illness [6, 14]. Mental illness among homeless individuals is also associated with unmet health care needs [16]. In addition, this population faces unique barriers to accessing primary care such as discrimination and difficulties with coordinating care [17]. These concerns highlight the need for primary care retention among homeless individuals with mental illness.

In recent years, concerted efforts have been made to house chronically homeless individuals using the Housing First approach [18]. In the Housing First model, individuals who are homeless and have mental illness receive immediate access to permanent housing in scattered-site independent units through the use of rent subsidies, in conjunction with portable mental health supports, without any requirement that they accept psychiatric treatment or abstain from substance use [19, 20]. Studies of Housing First have shown this approach to be effective in housing homeless individuals and, in some cases, reducing avoidable emergency department use, hospital admissions, and stays at correctional facilities [21–24].

Given the encouraging results from Housing First interventions in the US, the Mental Health Commission of Canada funded the At Home/Chez Soi Research Demonstration Project in Homelessness and Mental Health (hereafter referred to as the At Home Project) [25]. The At Home Project is the largest randomized controlled trial of Housing First conducted to date and investigates the effectiveness and cost-effectiveness of Housing First interventions in addressing the needs of homeless individuals with mental illness.

While previous research suggests that Housing First may lead to improved access to healthcare services by homeless individuals with mental illness [21], the relationship between Housing First and primary care retention among homeless individuals has not been previously studied. The main objective of this study was to assess the impact of a Housing First intervention on primary care retention among homeless individuals with mental illness. The secondary objective was to characterize associations between health status, medical comorbidities, and length of homelessness on primary care retention.

## Methods

### Study design and participants

Full details about the study design and recruitment procedures have been previously reported [25]. The At Home Project was a pragmatic, multi-site, randomized controlled trial of a Housing First intervention for homeless individuals with mental illness that took place in five cities across Canada (Vancouver, Winnipeg, Toronto, Montreal, and Moncton). The participants for the current study were homeless adults with mental illness and were selected from among the 575 individuals enrolled in the Toronto site of the project from October 2009 to July 2011. These participants were stratified according to severity of their psychiatric problems into High Needs or Moderate Needs groups. Those in the High Needs group were randomized into Housing First and Assertive Community Treatment (ACT) or Treatment As Usual (TAU), while those with Moderate Needs were randomized to Housing First and Intensive Case Management (ICM) or TAU [25].

A random sample of 200 participants (100 assigned to the Housing First intervention and 100 assigned to TAU) were selected for this prospective chart review study. Primary care medical records for these participants were reviewed. Medical records were used to determine if participants met criteria for retention in primary care in the 12 month period preceding randomization and the 12 month period thereafter.

### Interventions

ACT services were provided by a resource-intensive interdisciplinary team which included a psychiatrist, nurse, and other allied health professionals and teams typically had smaller caseloads. ICM offered less-intensive services provided by individual case managers who helped connect clients to various community resources.

All participants randomized to the intervention group received a rental allowance of $600 per month which was directly paid to the landlord. However, participants were named on the lease and entitled to all rights and obligations as a tenant under provincial legislation. Housing was provided in scattered-site private market apartments. Participants who were randomized to ACT or ICM had the opportunity to be connected to a family physician through their case manager. Intervention group participants were asked to meet with a project case manager at least once per week but participation in primary and mental health care was voluntary.

### Treatment as usual

Participants randomized to TAU were able to access a variety of pre-existing programs and services in the city of Toronto. TAU participants were provided with information about availability of these services and directed to both mainstream and homeless-specific health services.

### Data collection

During the enrollment process, each participant was asked where they had received any primary or ambulatory care in the previous 18 months. From these records, a database was created that listed all general practitioners, psychiatrists, community health centers and outpatient clinics utilized by participants in the past one and a half years. If specific contact information for the physician was not provided by the participant during enrollment, the College of Physicians and Surgeons of Ontario online directory was used to obtain this information. Members of the research team contacted each participant’s physicians or primary care clinics by telephone. Up to five attempts were made to contact each office. The physician’s office was provided with a copy of the participant’s consent for the release of medical records to the research team.

Chart reviews were conducted between January and August 2012. For each participant, the chart review was conducted at least one year after randomization. Medical records were obtained as photocopies or in electronic format for review by the research team. When necessary, a research team member reviewed the medical record at the physician’s office or clinic.

Data were extracted for a 24-month period that consisted of the 12 month period prior to randomization and the 12-month period following randomization. Charts were organized and reviewed using a data intake tool constructed during the study pilot phase. When examining medical charts, physical and mental health diagnoses were noted, as well as substance abuse. Diagnoses were classified as present or suspected according to the participant’s primary medical care record. Dates of visits to the family physician during the study period were also recorded to determine retention status. Research team members had access to identifying information for purposes of verifying the identity of participants during the chart review process. No identifying information was collected during the chart review process. Research team members who abstracted data from charts were also blinded to the participant’s allocated treatment group. To ensure reliability, 10% of charts were randomly sampled and reviewed independently by two research team members.

Self-reported overall health status was measured at baseline using the visual analogue scale section of the EQ5D, which is described by the EuroQol group [26]. Individuals were asked to indicate how they felt about their overall health on a visual scale from 0 to 100, where 0 is very poor health and 100 is excellent health. Number of comorbid diseases present was determined using a list of common conditions administered during baseline interviews.

### Data analysis

The pre-specified primary outcome was primary care retention in the year following randomization into the At Home Project. Retention was defined as having at least one visit with the same primary care provider in each of two consecutive six-month periods. Participants who had more than one PCP were considered retained if they met this definition with at least one of their providers. In a post-hoc analysis, the change in primary care retention from the year preceding randomization to the year following randomization was calculated.

Characteristics assessed for association with primary care retention were treatment assignment, need level, age, sex, ethno-racial status, Aboriginal status, language, education, marital status, employment, time spent homeless, longest period of homelessness, self-reported overall health status, and number of comorbid diseases. Univariate logistic regression models were used to determine if there was an association between each of the candidate predictors and primary care retention status while controlling for pre-randomization retention status. Predictors that were significant at p<0.20 or were clinically relevant were entered into the multivariate model in a block fashion. The variables included in the multivariate model included age, education and comorbidities. Pre-randomization retention status and treatment group were adjusted for in the final multivariate model. The goodness of fit of each model was tested using the Hosmer-Lemeshow test. All analyses were performed using SAS software version 9.3 (SAS Institute, Cary, NC).

### Consent and ethics approval

All study participants provided written informed consent. Capacity to consent was presumed if there were no concerns about the participant’s ability to understand and appreciate the informed consent process. However, when capacity of the participant was in question, capacity was assessed by a trained interviewer and if the person was deemed incapable of consent, they were not enrolled in the study. The study was approved by the Research Ethics Board of St. Michael’s Hospital in Toronto (REB #09–208), and registered with the International Standard Randomized Control Trial Number (ISRCTN 42520374).

## Results

**Fig 1:**
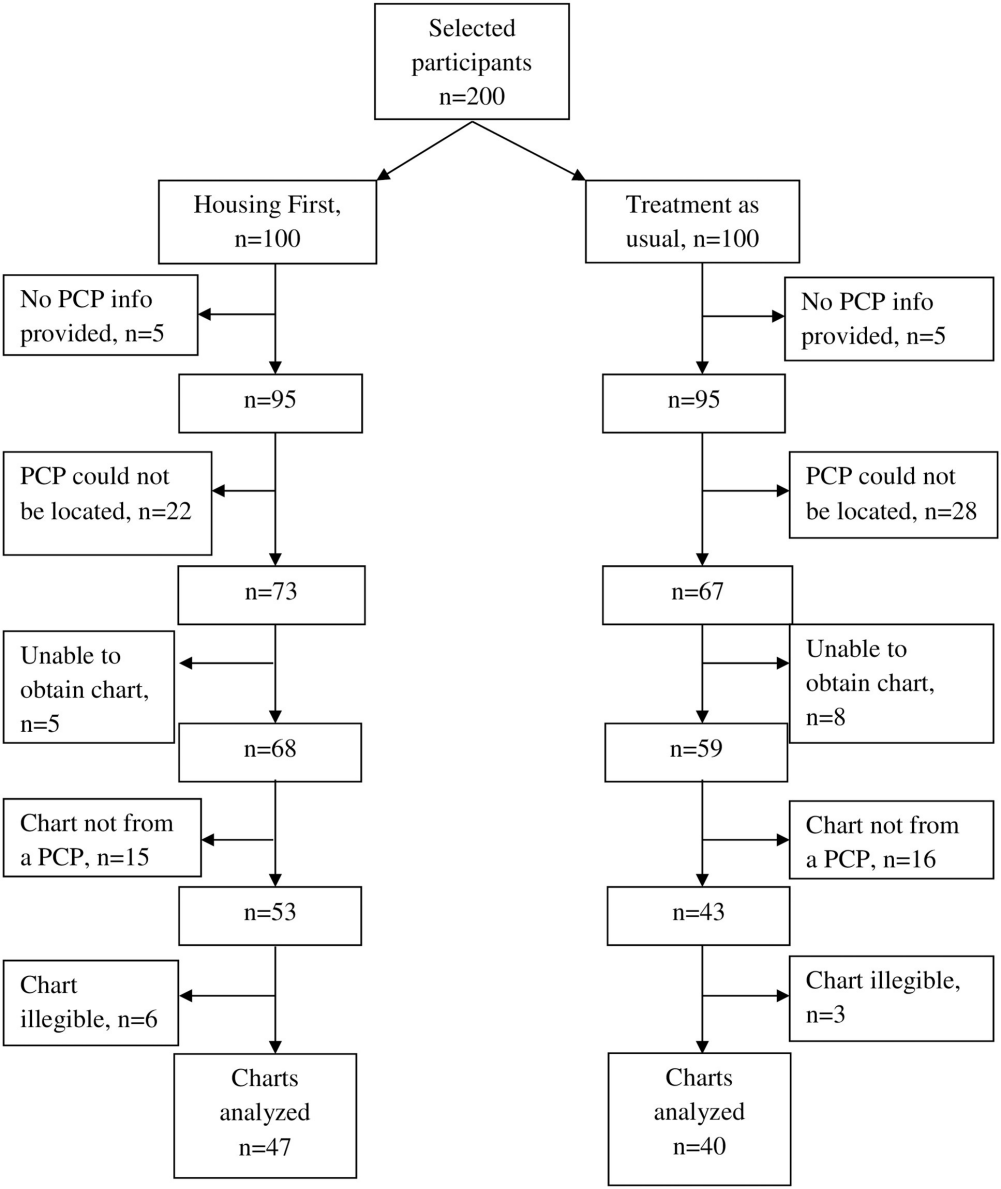
Flowchart of study participants included in chart review. Note: Abbreviations: PCP = primary care provider.

Charts were obtained for 47 of 100 participants who had been randomized to Housing First and 40 of 100 participants who had been randomized to TAU (p = 0.39 for the difference in proportion of charts obtained). In total, 87 charts were reviewed, with 70 coming from a private doctor’s office, 8 from community health centres, 6 from outpatient clinics, and 3 from clinics at drop-in centres for homeless persons (Fig 1). Charts could not be reviewed for participants who did not provide information on their PCP, whose PCP or chart could not be located, who identified a provider who was not a PCP, or whose charts were illegible (Fig 1).

Table 1 shows the characteristics of participants in the Housing First and TAU groups for whom chart review data was obtained. Overall, study participants spent an average of 77 months homeless, while the average length of their longest period of homelessness was approximately 41 months. Among this population, 53% of participants had more than four comorbid diseases.

**Table 1:** Characteristics of 87 participants randomized to Housing First or treatment as usual.

Characteristic	Housing First (n = 47)	Treatment As Usual (n = 40)	p-value
	N (%)	N (%)	
Need Level			
High Need	19 (40)	13 (33)	0.44
Moderate Need	28 (60)	27 (68)	
Gender			
Male	32 (68)	27 (68)	0.95
Not male	15 (32)	13 (33)	
Age Group			
< 40 years	23 (49)	17 (43)	0.55
≥ 40 years	24 (51)	23 (58)	
Ethno-racial			
Yes	23 (49)	21 (53)	0.74
No	24 (51)	19 (48)	
Language			
English	34 (72)	25 (63)	0.33
Other	13 (28)	15 (38)	
Education			
Less than high school	28 (60)	22 (55)	0.18
High school	6 (13)	11 (28)	
More than high school	13 (28)	7 (18)	
Marital Status			
Married/common-law	3 (7)	0	0.10
Single/divorced/ separated	43 (93)	40 (100)	
Employment			
Employed	3 (6)	0	0.10
Unemployed	44 (94)	40 (100)	
Alcohol abuse/dependence			
Yes	21 (45)	17 (43)	0.84
No	26 (55)	23 (58)	
Drug abuse/dependence			
Yes	24 (51)	17 (43)	0.43
No	23 (49)	23 (58)	
Lifetime duration of homelessness			
< 3 years	23 (49)	15 (38)	0.28
≥ 3 years	24 (51)	25 (63)	
Longest period of homelessness			
< 2 years	26 (55)	19 (48)	0.47
≥ 2 years	21 (45)	21 (53)	
Number of Comorbid Illnesses			
< 4	27 (57)	14 (35)	0.04
≥ 4	20 (43)	26 (65)	
EQ5D Score (Mean, SD)	62.7 (22.1)	63.5 (26.4)	0.89

There were 18 individuals in the Housing First intervention group and 19 individuals in the TAU group who were retained in primary care over the year following randomization (Table 2). As shown in Table 2, baseline rates of primary care retention in the year preceding randomization were different in the two groups, with higher pre-randomization primary care retention rates in the TAU group. The change in primary care retention rates from the year preceding randomization to the year following randomization was +10.6% in the Housing First group and -5.0% in the TAU group.

**Table 2:** Primary care retention in 87 participants in the Housing First or treatment as usual groups in the one year period preceding and following randomization.

Characteristic	Housing First (n = 47)	Treatment As Usual (n = 40)
	N (%)	N (%)
Retained Post-randomization		
Yes	18 (38)	19 (48)
No	29 (62)	21 (53)
Retained Pre-randomization		
Yes	13 (28)	21 (53)
No	34 (72)	19 (48)

While controlling for pre-randomization status in the univariate models, the only variable significantly associated with retention was the count of comorbid diseases (Table 3). The number of comorbid diseases was the only statistically significant variable to be added to the multivariate model from the univariate results. Although not statistically significant at p<0.05, age and education met the inclusion criteria and were added to the multivariate model as well. None of the characteristics included into the final multivariate model were significant at p<0.05 when controlling for pre-randomization retention status and treatment group. For the multivariate model, goodness of fit was demonstrated by the area under the curve (95% CI) = 0.73 (0.62, 0.84). The final model goodness of fit was as follows: Likelihood Ratio Test—Chi-sq = 14.70 df = 6, p = 0.02.

**Table 3:** Univariate and multivariate models for retention status.

Characteristic	Univariate Model	p-value	Multivariate Model	p-value
Retained Pre-randomization				
Yes	Controlled for in each univariate model		2.50 (0.91, 6.87)	0.08
No			1	
Treatment				
Housing First	0.84 (0.34, 2.07)	0.71	0.97 (0.35, 2.71)	0.96
Treatment As Usual	1		1	
Need Level				
High Need	0.75 (0.29, 1.97)	0.56		
Moderate Need	1			
Gender				
Male	0.82 (0.33, 2.09)	0.68		
Not male	1			
Age Group				
40 years and older	2.19 (0.89, 5.37)	0.09	1.64 (0.59, 4.53)	0.34
Less than 40	1		1	
Ethno-racial				
Yes	1.25 (0.52, 2.98)	0.62		
No	1			
Language				
English	1.45 (0.56, 3.78)	0.45		
Other	1			
Education				
More than high school	0.97 (0.33, 2.87)	0.37	1.03 (0.32, 3.29)	0.40
High school	0.34 (0.10, 1.23)	0.01	0.39 (0.10, 1.53)	0.16
Less than high school	1		1	
Alcohol abuse/dependence				
Yes	0.76 (0.31, 1.86)	0.55		
No	1			
Drug abuse/dependence				
Yes	1.28 (0.53, 3.11)	0.58		
No	1			
Time spent homeless				
3 years or more	1.22 (0.50, 2.94)	0.66		
Less than 3 years	1			
Longest period of homelessness				
2 years or more	1.22 (0.51, 2.92)	0.65		
Less than 2 years	1			
Co-morbidities				
≥ 4	3.61 (1.43, 9.13)	0.01	2.74 (0.95, 7.90)	0.06
< 4	1		1	
EQ VAS Overall Health Mean	0.99 (0.98, 1.01)	0.55		

## Discussion

Ongoing primary care is extremely important for individuals with mental illness. The Housing First model is highly effective in allowing homeless individuals with mental illness to achieve stable housing, but the effect of this model on primary care retention has not been well defined. Our study found that a Housing First intervention was not associated with a significant impact on primary care retention over one year of follow-up, compared to treatment as usual. The reasons for this observation are not entirely clear. Both the Housing First and TAU groups may have experienced similar barriers to obtaining primary care such as lack of availability and location of a primary care provider, lack of transportation, and discrimination [7]. It is possible that the case management services provided as part of the Housing First intervention did not prioritize connecting study participants with a primary care provider and thus had no effect on retention. Of note, the significant imbalance between the Housing First and TAU groups in their pre-randomization retention rates in primary care may have affected our results. Our finding that the change in primary care retention rates was higher in the Housing First group than in the TAU group suggests that Housing First may in fact have improved primary care retention.

The findings from the study add to a growing body of literature on interventions to improve primary care access among homeless adults [27]. These include outreach programs and an integrated primary care clinic within an outpatient treatment center which have led to improved preventive care and reduced emergency department use [28, 29]. Several studies have assessed the effect of housing and support services on primary care utilization. In one study of 385 homeless adults, intervention group participants who received housing and intensive case management services were more likely to have a primary care provider and a higher number of outpatient visits compared to the control group during the 2 year follow-up period [30].

Health status as measured by the EQ5D and duration of homelessness were not associated with retention. The presence of comorbidities was not associated with likelihood of being retained in primary care in the final multivariate model. This finding builds on the analyses of one prior study which found that a large proportion of homeless individuals with chronic medical conditions did not have access to primary care [7].

This study has several limitations. Primary care provider information was collected by self-report, so inaccurate recall may have resulted in the retrieval of fewer medical charts. In addition, a number of participants often indicated that their only health care provider was a psychiatrist and not a family physician, and these charts were excluded because these physicians were not considered to be primary care providers. In addition, the study only assessed primary care retention for a subset of Toronto site participants and difficulties in contacting providers and obtaining medical records resulted in a smaller than anticipated sample size. Thus, our study may have been underpowered to detect a difference between the Housing First and TAU groups. It is possible that there may have been differences in primary care retention between individuals who were randomized to ACT or ICM, but this analysis was not performed and would have been limited by the small sample size. Charts were reviewed for the one year period following randomization, and it is possible that more than one year of follow-up is required to observe improvements in primary care retention as the result of a Housing First intervention. Post-randomization eligibility based on chart availability might have also created an additional selection bias. As this study included a selected group of participants who accurately reported a primary care provider at baseline, this excluded participants whose engagement in primary care was unclear. As a result, the study did not capture primary care engagement for these individuals who may have been retained in primary care after obtaining housing.

Although several studies have assessed retention in individuals with addictions [31, 32], mental illness [33], and HIV [11, 34], few studies have assessed interventions to improve primary care retention for homeless and vulnerably housed populations. Challenges associated with defining and measuring retention, which have led to heterogeneous results across studies, also need to be addressed [35, 36]. In addition, interventions such as outreach and support services could be developed to improve the low rates of primary care retention among homeless populations [37, 38].

One possible approach to improve primary care retention could be facilitated by case managers who could help clients with finding a primary care provider and arranging subsequent health visits.

## Conclusion

Among homeless individuals with mental illness, a Housing First intervention that provided immediate housing and case management services did not have a significant effect on retention in primary care over a one-year follow-up period. Primary care retention remained low in both the Housing First and TAU groups. The findings of this study suggest that the Housing First model may need to place a greater emphasis on the importance of connecting clients with primary care providers, particularly for those with a higher number of medical comorbidities, in addition to providing housing and mental health supports.
